# Xanthatin Induces Cell Cycle Arrest at G2/M Checkpoint and Apoptosis via Disrupting NF-κB Pathway in A549 Non-Small-Cell Lung Cancer Cells

**DOI:** 10.3390/molecules17043736

**Published:** 2012-03-26

**Authors:** Lei Zhang, Junshan Ruan, Linggeng Yan, Weidong Li, Yu Wu, Li Tao, Feng Zhang, Shizhong Zheng, Aiyun Wang, Yin Lu

**Affiliations:** 1 Department of Clinical Pharmacy, College of Pharmacy, Nanjing University of Chinese Medicine, Nanjing 210029, China; 2 Department of Pharmacy, Anhui Provincial Hospital, Hefei 230001, China; 3 Jiangsu Key Laboratory for Pharmacology and Safety Evaluation of Chinese Materia Medica, Nanjing University of Chinese Medicine, Nanjing 210029, China

**Keywords:** xanthatin, non-small-cell lung cancer, cytotoxicity, cell cycle, apoptosis

## Abstract

Xanthatin, a natural sesquiterpene lactone, has significant antitumor activity against a variety of cancer cells, yet little is known about its anticancer mechanism. In this study, we demonstrated that xanthatin had obvious dose-/time-dependent cytotoxicity against the human non-small-cell lung cancer (NSCLC) cell line A549. Flow cytometry analysis showed xanthatin induced cell cycle arrest at G2/M phase. Xanthatin also had pro-apoptotic effects on A549 cells as evidenced by Hoechst 33258 staining and annexin V-FITC staining. Mechanistic data revealed that xanthatin downregulated Chk1, Chk2, and phosphorylation of CDC2, which contributed to the cell cycle arrest. Xathatin also increased total p53 protein levels, decreased Bcl-2/Bax ratio and expression of the downstream factors procaspase-9 and procaspase-3, which triggered the intrinsic apoptosis pathway. Furthermore, xanthatin blocked phosphorylation of NF-κB (p65) and IκBα, which might also contribute to its pro-apoptotic effects on A549 cells. Xanthatin also inhibited TNFα induced NF-κB (p65) translocation. We conclude that xanthatin displays significant antitumor effects through cell cycle arrest and apoptosis induction in A549 cells. These effects were associated with intrinsic apoptosis pathway and disrupted NF-κB signaling. These results suggested that xanthatin may have therapeutic potential against NSCLC.

## 1. Introduction

Cancer remains a leading cause of death worldwide. Chemotherapy as a systemic drug-based approach is still irreplaceable in clinical contexts. Currently, natural products have been a primary source of chemotherapeutics and have provided abundant candidates with promising antitumor activities [1]. Sesquiterpene lactones (SLs) are a group of natural terpenoids exhibiting a wide range of bioactivities, including antimicrobial, cytotoxic, antiproliferative and antiinflammatory effects [2]. Many SLs such as artemisinin, thapsigargin and parthenolide and many of their derivatives are emerging as potent anticancer agents in cancer chemotherapy and chemoprevention [3,4]. They are found to be selective toward tumors by targeting specific signaling pathways, making them lead compounds in cancer clinical investigations [4].

Xanthatin is a bicyclic sesquiterpene lactone isolated mainly from *Xanthium* plants (Asteraceae). Recent studies demonstrated that xanthatin had significant antitumor activity in a variety of cell culture systems implicated in colon, breast, lung, cervix and skin cancers [5,6]. However, the molecular mechanisms underlying these effects remain poorly understood. It is known that tumor cell survival, death and cell cycle are interconnected mechanistically [7]. Molecular associations between these events give high possibility to develop pharmacological agents that can block cell proliferation pathways and drive them into apoptosis. Given the potent antitumor effects of xanthatin, we presumed that xanthatin could arrest cell cycle and induce apoptosis in tumor cells. Nuclear factor-kappa B (NF-κB) is a transcription factor critical for controlling cell proliferation and apoptosis [8]. It is reported that SLs are a potential source of NF-κB inhibitors [4]. Moreover, xanthatin was shown to inhibit NF-κB activity in activated microglia [9]. Thus we hypothesized that xanthatin could disrupt NF-κB signaling in tumor cells, leading to cell growth blockade and apoptosis.

We here selected the non-small-cell lung cancer (NSCLC) cell line A549 to investigate the antitumor role of xanthatin, because this cell line is typically malignant and invasive, and is p53 wild-type. Transcription factor p53 is a tumor suppressor critically involved in many cellular events including cell cycle control and apoptosis [10]. In the present studies, we demonstrated that xanthatin potently inhibited cell viability and induced cell cycle arrest at G2/M checkpoint and apoptosis in A549 cells. These effects were associated with activation of p53 and inhibition of NF-κB signaling, leading to decreased Bcl-2/Bax ratio and activated caspase cascade. These results indicated that xanthatin could be exploited as a promising candidate for treatment of lung cancer.

## 2. Results and Discussion

### 2.1. Results

#### 2.1.1. Xanthatin Inhibited A549 Cell Growth Dose-/Time-Dependently

**Figure 1:**
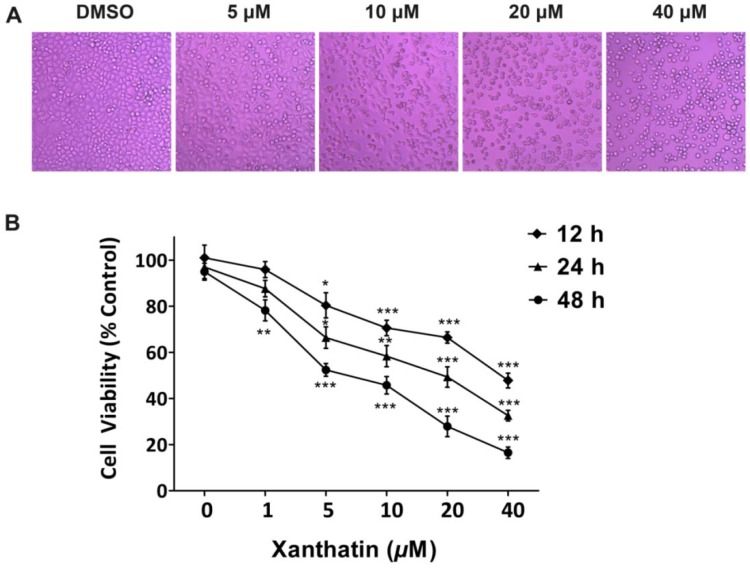
Cytotoxic effects of xanthatin on A549 cells. (**A**) Cell morphology under light microscopy after incubation with xanthatin at indicated concentrations for 24 h (×200); (**B**) Inhibitory effects of xanthatin on the cell viability of A549 cells by MTS assay. Data were presented as means ± SD by three independent experiments. Significance: * *p* < 0.05 *versus* the control; ** *p *< 0.01 *versus* the control, *** *p* < 0.001 *versus* the control.

To determine the cytotoxic effects of xanthatin on A549 cells, we first evaluated the alterations in cell morphology. The results showed that xanthatin led to apparent morphological changes in a dose-dependent manner. The conspicuous changes observed in xanthatin-treated cells included cell shrinkage, roundup and extensive detachment of the cells from the culture substratum. These changes became increasingly visible with dose increased, but were absent in the control cells (Figure 1A). We subsequently used MTS assay to determine xanthatin effects on A549 cell growth at different intervals. The data showed that xanthatin had inhibitory effects on A549 cell growth both dose- and time-dependently (Figure 1B). After 12 h treatment, xanthatin at 5 μM inhibited cell growth significantly compared with the control (*p* < 0.05). The IC_50_ values of xanthatin inhibition of A549 cell growth at 12, 24 and 48 h were 36.2, 21.1 and 8.3 μM, respectively.

#### 2.1.2. Xanthatin Induced Cell Cycle Arrest at G2 Phase in A549 Cells

**Figure 2:**
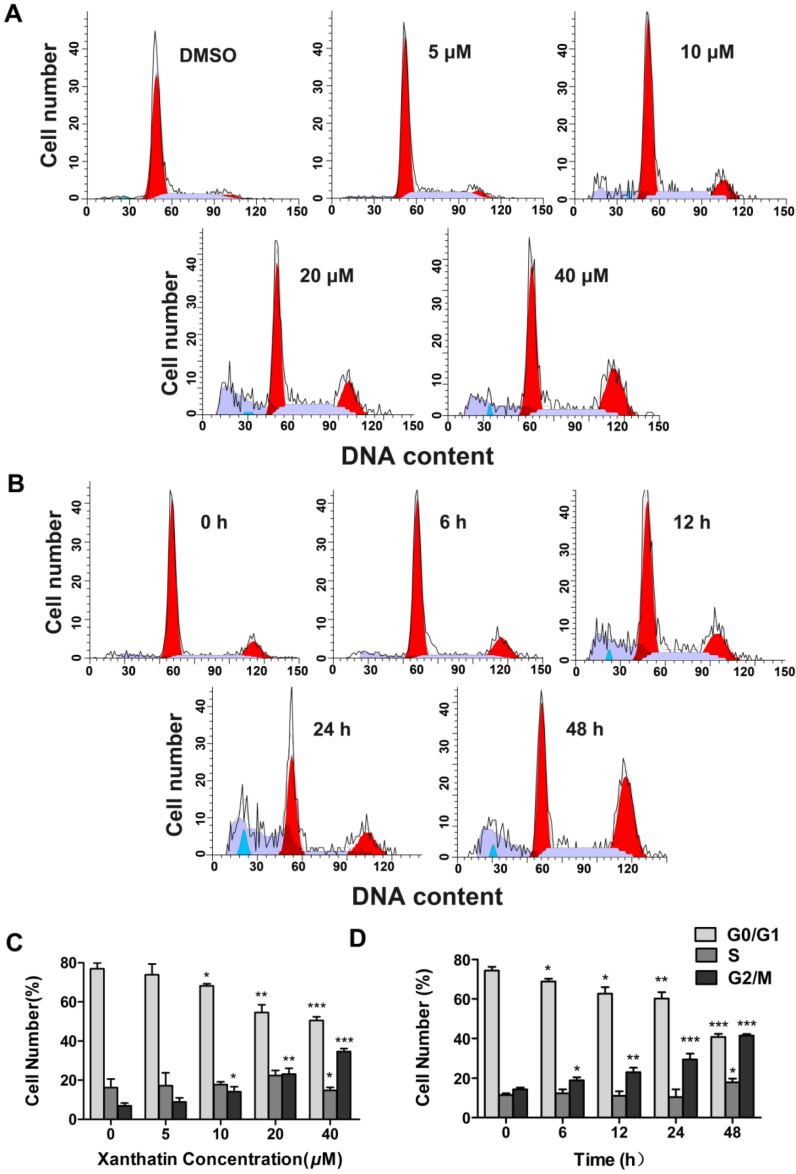
Xanthatin induced G2 phase cell cycle arrest in A549 cells. (**A**) Representative histograms depicting cell cycle distribution in A549 cells treated with xanthatin at indicated concentrations for 24 h; (**B**) Representative histograms depicting cell cycle distribution in A549 cells treated with xanthatin (40 μM) for indicated time; (**C**) The cell cycle distributions are presented as cumulative proportions of cells within each of three cell cycle compartments (G0/G1, S and G2/M) in A549 cells treated with xanthatin at indicated concentrations for 24 h; (**D**) The cell cycle distributions are presented as cumulative proportions of cells within each of three cell cycle phases (G0/G1, S and G2/M) in A549 cell cultures treated with xanthatin (40 μM) for indicated time. Data were presented as means ± SD by three independent experiments. Significance: * *p* < 0.05 *versus* the control; ** *p* < 0.01 *versus* the control, *** *p* < 0.001 *versus* the control.

**Figure 3:**
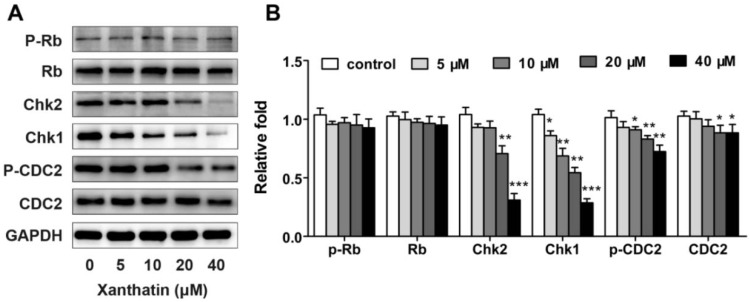
Western blot analysis of cell cycle-related proteins in A549 cells treated with xanthatin. (**A**) The protein levels of G2/M cell cycle regulatory molecules including P-Rb, Rb, chk1, chk2, CDC2, p-CDC2 in A549 cells treated with xanthatin at indicated concentrations for 24 h were analyzed by western blot assay; (**B**) Densitometry analysis of the protein levels of G2/M cell cycle regulatory molecules. Data were presented as means ± SD by three independent experiments. Significance: * *p* < 0.05 *versus* the control; ** *p* < 0.01 *versus* the control, *** *p* < 0.001 *versus* the control.

We next tested whether xanthatin could affect the cell cycle progression in A549 cells via flow cytometric analysis. The results showed that exposure of A549 cells to xanthatin resulted in a significant increase in the G2 phase accompanied by a decreased distribution in the G1 phase dose- and time-dependently (Figure 2A,B). Cells showed more striking G2/M arrest from 6.86% in vehicle-treated cells to 29.61% in the experimental group at 24 h (Figure 2C). Treatment with xanthatin at 40 μM for 48 h resulted in 42.42% of A549 cells in the G2/M phase, compared with 14.24% of vehicle-treated cells at 0 h. The percentage of G2 phase increased by 2.98-fold on treatment of A549 cells with 40 μM xanthatin compared with the control (Figure 2D). It is known that cell cycle progression is controlled by key checkpoint enzymes. Our further data showed that xanthatin dose-dependently downregulated the expressions of Chk1, Chk2, CDC2 and p-CDC2, which are key proteins related to the G2 phase cell cycle checkpoint. However, the key regulator for G1 checkpoint Rb and its phosphorylation were not affected by xanthatin (Figure 3A,B). Collectively, these findings demonstrated that xanthatin induced cell cycle arrest at G2 phase via decreasing the expression and phosphorylation of key checkpoint proteins in A549 cells.

#### 2.1.3. Xanthatin Induced Apoptosis via p53 Activation and Intrinsic Pathway in A549 Cells

**Figure 4:**
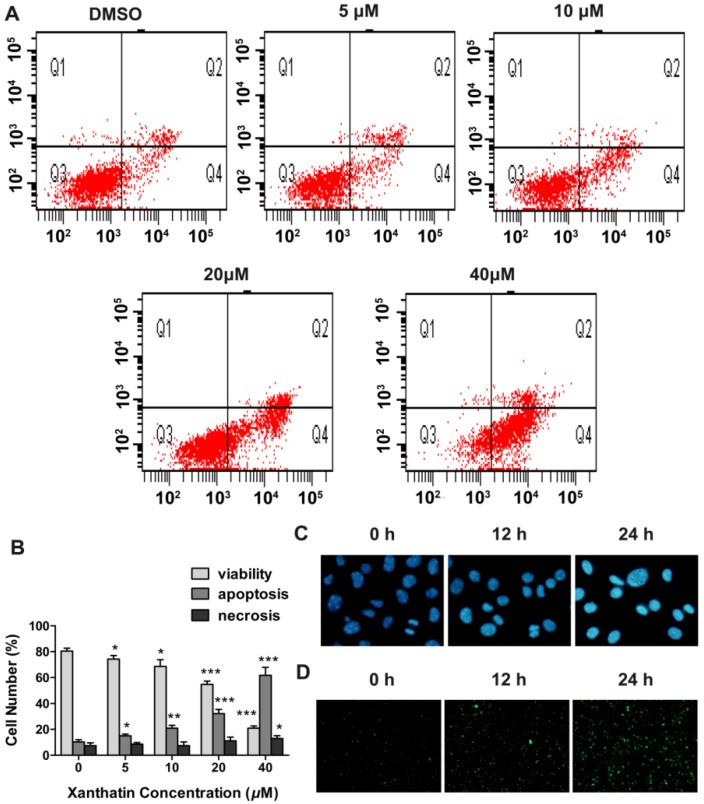
Xanthatin induced apoptosis in A549 cells. (**A**) Flow cytometric analysis of annexin V-FITC/PI double-staining in A549 cells treated with xanthatin at indicated concentrations for 24 h; (**B**) The percentages of cells within each of the cell stage in A549 cells treated with xanthatin at indicated concentrations for 24 h; (**C**) Cell morphology under fluorescence microscopy by Hoechst 33258 staining in A549 cells treated with xanthatin (20 μM) for indicated time (×400); (**D**) Cell morphology under fluorescence microscopy by Annexin V-FITC staining in A549 cells treated with xanthatin (20 μM) for indicated time (×100). Data were presented as means ± SD by three independent experiments. Significance: * *p* < 0.05 *versus* the control; ** *p* < 0.01 *versus* the control, *** *p* < 0.001 *versus* the control.

We then investigated whether the inhibited cell viability induced by xanthatin was associated with apoptosis via double staining of the cultures with PI and annexin V-FITC. As shown in Figure 4A, we observed an increase in early apoptotic (annexin V^+^/PI^−^) cells dose-dependently in the presence of xanthatin. The quantitative analysis showed that xanthatin at 5 μM began to induce significant apoptosis in A549 cells (Figure 4B). We also observed that the number of apoptotic cells increased with the increasing treatment time of xanthatin at 20 μM especially at 12 and 24 h (Figure 4D), suggesting the apoptosis-inducing effects of xanthatin was also time-dependent. Moreover, Hoechst 33258 staining assay showed that morphological alterations were caused in the nucleus of xanthatin-treated A549 cells at different time points. Xanthatin led to smaller nuclei with brilliant blue staining (Figure 4C). These results provided additional evidence for xanthatin-induced apoptosis in A549 cells.

**Figure 5:**
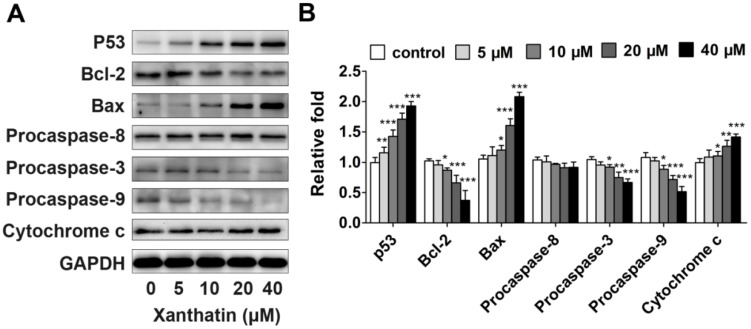
Western blot analysis of apoptosis-related proteins in A549 cells treated with xanthatin. (**A**) Protein expression of p53, Bax, Bcl-2, procaspase-8, procaspase-3 and procaspase-9 in A549 cells treated with xanthatin at indicated concentrations for 24 h. GAPDH was used as the control; (**B**) Densitometry analysis of the protein levels of apoptosis-related proteins in A549 cells treated with xanthatin. Data were presented as means ± SD by three independent experiments. Significance: * *p* < 0.05 *versus* the control; ** *p* < 0.01 *versus* the control, *** *p* < 0.001 *versus* the control.

To further elucidate the underlying mechanisms, we first detected the expression of p53, a tumor suppressor playing a crucial role in cell cycle progression, apoptosis and repair [10], in A549 cells treated with xanthatin. The data showed that xanthatin upregulated the expression of p53 compared to the control (Figure 5A,B). Furthermore, apoptosis can be triggered through two signaling pathways: the extrinsic pathway (death receptor pathway) and the intrinsic pathway (the mitochondrial pathway) [11]. The intrinsic apoptotic pathway hinges on the balance of activity between pro- and anti-apoptotic members of the Bcl-2 superfamily proteins [12]. Caspases, especially caspase-3, are the chief effectors in apoptosis [13]. Our data here demonstrated that xanthatin dose-dependently decreased Bcl-2 levels and increased Bax levels, and promoted the cleavage of caspase-3, whereas had no effects on expression of caspase-8 (Figure 5A,B), which is a key mediator of the extrinsic pathway. The data also showed that xanthatin could induce the release of cytochrome c, which is a hallmark of mitochondrial dysfunction. These results indicated that xanthatin disrupted the balance between Bax and Bcl-2 contributing to apoptosis via caspase activation in the mitochondria-dependent pathway rather than the extrinsic pathway in A549 cells. Taken together, Xanthatin activated p53 expression, which subsequently induced apoptosis through upregulating Bax/Bcl-2 ratio, then evoking cytochrome c release and triggering the apoptotic caspase cascade such as activated caspase-9 and caspase-3.

#### 2.1.4. Xanthatin Disrupted NF-κB Signaling in A549 Cells

**Figure 6:**
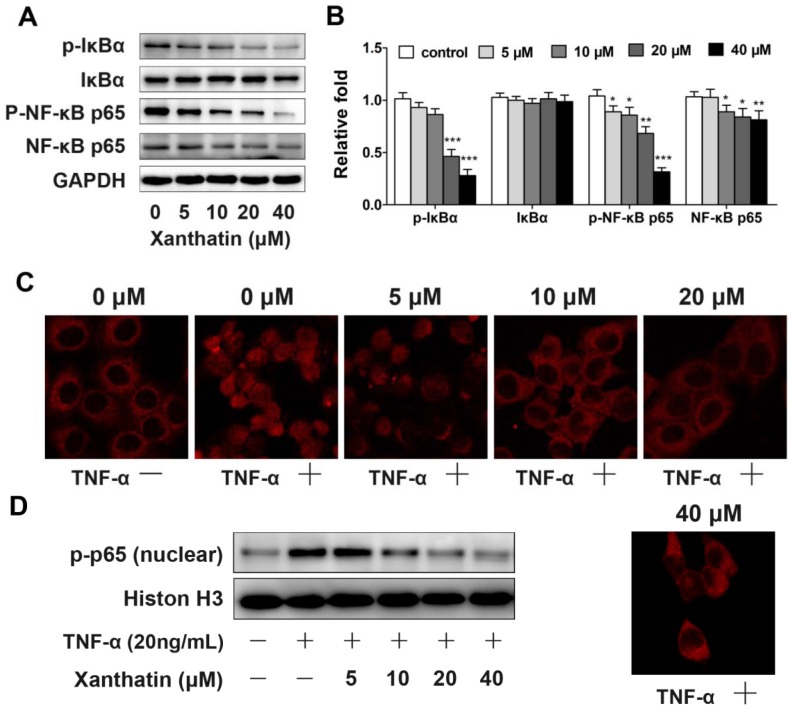
Xanthatin disrupted NF-κB signaling in A549 cells; (**A**) Protein expression of p-IκBα, IκBα, p-NF-κB p65, NF-κB p65 in A549 cells treated with xanthatin at indicated concentrations for 24 h by Western blot analysis. GAPDH was used as the control; (**B**) Densitometry analysis of NF-κB pathway related proteins in A549 cells treated with xanthatin; (**C**) Fluorescence cells were photographed under a laser scanning confocal microscope by an immunocytochemical methodin A549 cells treated with xanthatin for 24 h and then 20 ng/mL TNFαfor 10 min(×400); (**D**) Nuclear extracts were analyzed by Western blotin A549 cells treated with xanthatin for 24 h and then 20 ng/mL TNFαfor 10 min. Histon H3 was used as reference.Data were presented as means ± SD by three independent experiments. Significance: * *p* < 0.05 *versus* the control; ** *p* < 0.01 *versus* the control, *** *p* < 0.001 *versus* the control.

NF-κB is a critical transcription factor that regulates the transcription of many genes associated with tumourigenesis [8]. Thus we investigated the involvement of this signaling in order to further define the molecular mechanisms underlying xanthatin-induced cell apoptosis. The data demonstrated that the phosphorylation of NF-κB (p65) was decreased by xanthatin. At the same time, the phosphorylation level of the inhibitory protein, IκBα, which binds to the NF-κB heterodimer (p50/p65) was downregulated by xanthatin (Figure 6A,B). These results suggested that xanthatin inhibited NF-κB signaling in A549 cells. The abrogated release of IκBα from IκBα/p50/p65 complex presumably caused decreased expression of target genes such as Bcl-2, and eventually induced apoptosis in A549 cells.

Western blot assay showed that xanthatin affect the IκBα/NF-κB signal pathway. In order to understand weather xanthatin’s pro-apoptotic effects in A549 cells were partly due to inhibition of translocation of NF-κB, we used an immunofluorescence method to visualize the dynamic movement of the NF-κB (p65) between the cytoplasm and nucleus. In this assay TNFα, a known NF-κB activator, was used. The majority of the p65 subunit was detected in the cytoplasm of A549 cells in control and xanthatin groups, but the addition of 20 ng/mL TNFα resulted in complete translocation of p65 into the nucleus and pretreatment with xanthatin inhibited TNFα-induced NF-κB (p65) translocation (Figure 6C). Analysis of the nuclear protein extracts by Western blot showed that xanthatin (10 μM) completely suppressed the TNFα-induced activation of p65 (Figure 6D). These results suggested that xanthatin inhibited NF-κB signaling in A549 cells by suppressing NF-κB (p65) translocation.

### 2.2. Discussion

The anticancer properties of SLs has attracted a great deal of interest. Various SLs have been shown to execute their antitumor capability via suppression of inflammatory responses, prevention of metastasis and induction of apoptosis in cell culture and animal models [3,4]. Xanthatin investigated here is an outstanding SL compound in terms of antitumor activity [5,6]. Moreover, xanthatin showed strong gastric protective activity [14] and exhibited little or no toxicity to animals, with an LD_50_ value of 800 mg/kg [15], so xanthatin may be a highly effective inhibitor of tumor cell proliferation. However, the molecular basis for this selectivity toward cancer cells remains to be defined. Until recently, there has been an investigation addressing the mechanisms underlying xanthatin’s antitumor effects in MDA-MB-231 breast cancer cells that were p53 mutant [16]. We herein selected A549 lung cancer cells that were p53 wild type to investigate xanthatin effects on cell cycle progression and apoptosis, and the involved molecular pathways. These findings could further explain the selectivity of xanthatin implicated in cancer chemotherapy.

Our study demonstrated that xanthatin selectively induced G2/M arrest in A549 cells, leading to decreased cell viability and growth in a dose- and time-dependent manner. It is known that cell growth is controlled by cell cycle progression, a highly regulated process [17,18]. The standard cell cycle is divided into four non-overlapping phases, namely G1, S, G2 and M phases in sequence. Each phase has checkpoints that cause cell cycle arrest and activation of repair mechanisms [19,20]. Unlike normal cells that rely on the G1 checkpoint to protect against DNA damage, cancer cells are more dependent on the G2 checkpoint for DNA damage repair [21]. These insights give rise to the concept that cell cycle G2 checkpoint could be a specific target for cancer therapy. Both Chk1 and Chk2 are essential components in the G2 checkpoint via phosphorylating Cdc25c in response to DNA damage [22]. Actually, much evidence has suggested that Chk2 and/or Chk1 inhibitors may be good candidates for therapeutic G2 checkpoint abrogation implicated in cancer chemotherapy [23,24]. Our present study demonstrated that xanthatin was such an agent that selectively induced G2/M arrest in A549 cells, which was attributed to downregulation of Chk1 and Chk2 and decreased phosphorylation of CDC2. Our findings were in agreement with the established molecular recognitions and strongly suggested that the antitumor properties of xanthatin were consistent with the criterion for developing selective agents for cancer treatment.

To further examine the underlying mechanisms, we investigated xanthatin’s effects on apoptosis. Drug-induced cell cycle arrest can cause inefficient repair, leading to survival of genomically unstable cancer cells, or apoptosis if the damage is unrepairable. To switch the balance toward cell death with simultaneous abrogation of the DNA repair functions can enhance chemotherapy sensitization [25]. Some signaling molecules share function between cell proliferation and cell death machinery. We here found that xanthatin dose-dependently activated p53 concomitant with significant apoptosis in A549 cells. The mechanistic data indicated that p53 activation by xanthatin upregulated the ratio of Bax/Bcl-2, and subsequently promoted procaspase-9 cleavage and activated caspase-3. Many studies have pointed to a central role for p53 in balancing proliferation and apoptosis [10]. As a primary component of the G2 checkpoint, p53 not only conducts DNA damage signals and suppresses G2/M transition, but also is required for apoptosis induction. Through its transcriptional activities, p53 regulates the balance between the proapoptotic genes like Bax and the antiapoptotic genes like Bcl-2 [26]. These events lead to caspase activation and apoptosis. Our present data suggested the involvement of p53-involved mitochondria apoptosis pathway in xanthatin’s effects. Moreover, we also ruled out the involvement of the extrinsic apoptosis pathway, because xanthatin had no effects on procaspase-8 expression. This probably accounted for xanthatin’s selectivity in the mode of action.

NF-κB is shown to stimulate cell survival and proliferation, and its increased activity is positively associated with many cancer types including lung cancer [27]. Thus to further gain insights into the mechanisms of xanthatin-induced apoptosis, we investigated the expressions of key proteins in NF-κB pathway. We demonstrated that xanthatin inhibited the phosphorylation of NF-κB p65 subunit. Xanthatin also inhibited TNFα induced NF-κB (p65) translocation. These effects could disrupt NF-κB translocation into the nucleus to transactivate the genes involved in cell proliferation. Disruption of NF-κB pathway by xanthatin contributed to its inhibitory effects on cell cycle progression and apoptosis induction in A549 cells. These molecular insights could make xanthatin a more valuable candidate for cancer chemotherapy given that invasion and metastasis of tumor cell are closely related to inflammation, while NF-κB is indeed a key player in inflammation-induced tumor metastasis. There is evidence that the basal NF-κB activity is often low or required for cell differentiation rather than oncogenesis [8]. This could be the reason why the agents derived from sesquiterpene lactones, shown to be potential NF-κB inhibitors, selectively target tumor cells [28]. Targeting NF-κB pathway via which inflammation contributes to tumor progression and metastasis could lead to innovative approach for treating cancer [27]. Our investigation demonstrated that xanthatin disrupted NF-κB signaling in A549 cells. To determine its precise antitumor role and associations with inflammatory system requires further investigations especially the *in vivo* evidence.

## 3. Experimental

### 3.1. Xanthatin Preparation

The whole plant of *Xanthium sibiricum *Patrin ex Widder. (*X. strumarium* L.) (Asteraceae) was collected in Xuzhou city of Jiangsu Province, China, in May 2009 and authenticated by Jianwei Chen (College of Pharmacy, Nanjing University of Chinese Medicine, Nanjing, China). Xanthatin was isolated and purified from air-dried aerial parts of *X. sibiricum* by our group as previously described [29] and the purity exceeded 95% as determined by a HPLC method. Xanthatin was dissolved in dimethyl sulfoxide (DMSO) for all experiments in this study.

### 3.2. Regents

RPMI-1640 medium and heat-inactivated fetal bovine serum (FBS) were purchased from Gibco (Carlsbad, CA, USA). Primary antibodies against p-Rb, p-CDC2, caspase-3, caspase-8, caspase-9, NF-κB, p-NF-κB, IκBα, p-IκBα were purchased from Cell Signaling Technology (Boston, MA, USA). Primary antibodies against CDC2, Chk1, Chk2, Rb, Bax, Bcl-2, Cytochrome C, GAPDH and Histon H3, goat anti-mouse IgG-HRP and goat anti-rabbit IgG-HRP were obtained from Bioworld (St. Louis Park, MN, USA). Primary antibody against p53 was obtained from Signalway Antibody (Jiangsu, China).

### 3.3. Cell Culture

The NSCLC cell line A549 (Chinese Academy of Science, Shanghai, China) was cultured in RPMI-1640 medium supplemented with 10% FBS, 100 U/mL penicillin and 100 μg/mL streptomycin. Cultures were maintained in a humidified atmosphere with 5% CO_2_ at 37 °C. Cells were passaged every 4–5 days.

### 3.4. Cell Morphology Assessment

A549 cells were cultured in RPMI-1640 medium until mid-log phase for experimental use. DMSO used as control in all experiments (0.5%) or xanthatin at indicated concentrations (final concentration of 5, 10, 20, 40 μM) were added to the culture medium. After incubation for 12, 24 and 48 h, images of the cell morphology were taken with an inverted microscope (Olympus IX-70, Tokyo, Japan).

### 3.5. MTS Assay

The effects of xanthatin on proliferation of A549 cells were assessed using the MTS [3-(4,5-dimethylthiazol-2-yl)-5-(3-carboxymethoxyphenyl)-2-(4-sulfophenyl)-2H-tetrazolium] assay. Briefly, A549 cells at mid-log phase were seeded in 96-well plates (5 × 10^3^ per well) in 200 μL of medium. After 24 h incubation, cells were exposed to 0.5% DMSO or xanthatin at indicated concentrations for 12, 24 and 48 h. Then, MTS (final concentration of 333 μg/mL)/PMS (25 μM) (Promega, Madison, WI, USA) was added and the cells were incubated for 2 h at 37 °C. The spectrophotometric absorbance was measured by SPECTRAmax microplate spectrophotometer (Molecular Devices, Sunnyvale, CA, USA) at 490 nm. Triplicate experiments were performed. The concentration of xanthatin resulting in 50% inhibition of control growth (IC_50_) was calculated using PASW Statistics 18 for windows.

### 3.6. Cell Cycle Analysis

Cell cycle analysis was performed as described previously [30]. Cells were seeded in growth medium in 6-well plates (3 × 10^5^ per well) and were grown overnight at 37 °C in a humidified incubator with 5% CO_2_. Cells were then treated with xanthatin at indicated concentrations for 6, 12, 24 and 48 h. They were harvested (including attached and detached cells) and fixed with 75% alcohol at 4 °C. Distribution of cells with different DNA content was analyzed using the Cellular DNA Flow Cytometric Analysis Kit (KeyGEN, Nanjing, China) according to the manufacture’s instructions. The percentage of cell cycle distribution was determined using a FACScan laser flow cytometer (Becton Dickinson, San Jose, CA, USA). The data were analyzed using the software CELLQuest.

### 3.7. Annexin-V/PI Double Staining Assay

A549 cells were treated with xanthatin at indicated concentrations for 12 or 24 h. Then they were harvested (including attached and detached cells), washed and resuspended with PBS. Apoptotic cells were determined with an FITC Annexin V Apoptosis Detection Kit (KeyGEN, Nanjing, China) according to the manufacturer’s protocol. Apoptosis was analyzed by fluorescence microscope and FACScan laser flow cytometer. The data were analyzed using software (CELLQuest).

### 3.8. Hoechst 33258 Staining Assay

After xanthatin treatment (20 μM) for 0, 12 or 24 h, attached cells were washed twice with PBS and fixed with 4% formaldehyde at 4 °C for 30 min. The fixing solution was removed and cells were washed twice with PBS before staining with Hoechst 33258 (Beyotime, Nanjing, China). After staining for 10 min, cells were washed again and observed under a fluorescence microscope (Olympus IX-70, Tokyo, Japan). At least 20 fields were randomly selected and images were taken.

### 3.9. Western Blot Analysis

Whole cell proteins were extracted using radioimmunoprecipitation (RIPA) assay buffer supplemented with 1 mM PMSF and 1:100 dilution of Protease Inhibitor Cocktail (Bestbio, Shanghai, China). Protein concentration was determined using the BCA™ protein assay kit (Pierce, Rockford, IL, USA). Protein samples (50 μg) were separated on SDS-polyacrylamide gels (SDS-PAGE) and transferred to a polyvinylidene difluoride (PVDF) membrane (Millipore, Bedford, MA, USA). After blocked with 5% nonfat dry milk in Tris-buffered saline (TBS) containing 0.1% Tween 20 for 2 h, the membranes were incubated with primary antibodies at 1:200 to 1,000 dilutions with 5% BSA in TBST overnight at 4 °C. The blots were washed and incubated with HRP-conjugated secondary antibodies (1:10,000, Bioworld, St. Louis Park, MN, USA) for 1 h at room temperature. Membranes were visualized using enhanced chemiluminescence (Immobilon ECL, Millipore).

### 3.10. NF-κB (p65) Translocation

The effect of xanthatin on TNFα-induced nuclear translocation of p65 was examined using an immunocytochemical method. Cells were seeded in growth medium in 24-well plates (5 × 10^4^ per well) and grown overnight at 37 °C in a humidified incubator with 5% CO_2_. Cells were pretreated with 40 μM xanthatin for 24 h before being stimulated with 20 ng/mL TNFα (Signalway, Jiangsu, China) for 10 min. Treated cells were washed with cold PBS followed by fixation with cold acetone for 10 min at 4 °C. Cells were permeabilized with 0.5% triton X-100 (Sigma, St. Louis, MO, USA) for 10 min at room temperature, washed with PBS and blocked with 1% bovine serum albumin (BSA) for 1 h. Rabbit anti-p65 antibody was added and incubated overnight at 4 °C. After washing with PBS for three times, goat anti-rabbit IgG-Tritc (ZHONGSHAN, Beijing, China) was added and incubated for 1 h at room temperature. Fluorescence cells were observed and photographed under a laser scanning confocal microscope (LEICA, Mannheim, Germany). The nuclear proteins after treating with xanthatin or/and TNF-α were extracted using Nuclear and Cytoplasmic Extraction Reagents (KeyGEN, Nanjing, China) following the instruction. The extracts were analyzed by Western blot assay using antibodies against p-NF-κB (p65). Histon H3 was used as reference.

### 3.11. Statistical Analysis

All data are expressed as mean ± SD. Statistical comparisons between groups performed using 1-way ANOVA followed by Student’s t-test and a *p* value of less than 0.05 was considered statistically significant.

## 4. Conclusions

In summary, we have demonstrated that xanthatin caused potent cell cycle arrest at the G2/M checkpoint and induced apoptosis in A549 lung cancer cells, leading to inhibited cell growth. The potential mechanisms involved p53-involved intrinsic apoptosis pathway and activation of caspase cascade. Moreover, xanthatin’s antitumor activity was also associated with disputed NF-κB signaling in A549 cells. Our findings provided novel insights into the role of xanthatin in lung cancer therapy.
